# TRANSITIONS BETWEEN SARCOPENIA STATES AND ITS DETERMINANTS IN COMMUNITY-DWELLING OLDER ADULTS

**DOI:** 10.1093/geroni/igad104.2775

**Published:** 2023-12-21

**Authors:** Miji Kim, Hyung Eun Shin, Chang Won Won

**Affiliations:** Kyung Hee University, Seoul, Republic of Korea; Kyung Hee University, Seoul, Republic of Korea; Kyung Hee University Hospital, Seoul, Republic of Korea

## Abstract

Sarcopenia is characterized by an age-related progressive reduction in muscle mass and function. Few observational studies have characterized the dynamic nature of sarcopenia, which is potentially reversible. However, little is known about the determinants of sarcopenia reversal among community-dwelling older adults. We aimed to explore transitions in sarcopenia status and identify factors associated with the reversibility of sarcopenia over a 2-year follow-up period. We conducted prospective analyses (n=1,992) among participants (mean age, 76.3±3.9 years; 47.6% men) who underwent measurement of sarcopenia status at baseline and 2-year follow-up from the Korean Frailty and Aging Cohort Study. Sarcopenia status was diagnosed using the criteria of the 2019 Asian Working Group for Sarcopenia. After 2 years of follow-up, 224 (11.2%) individuals developed sarcopenia, 176 (8.8%) had reversed their sarcopenia, 276 (13.9%) remained in a sarcopenic state, and 1,317 (66.1%) had maintained a non-sarcopenic state. For men, moderate to high levels of physical activity (odds ratio [OR]=2.50 [95% confidence interval (CI)]: 1.31–4.56), resistance training more than 2–3 times per week (OR=2.03, 95% CI: 1.04–3.97), and lower body mass index (OR=0.67, 95% CI: 0.55–0.82) were associated with greater odds reversing sarcopenia. For women, cognitive function (OR=1.12, 95% CI: 1.05–1.26 per 1-point increase in the Mini-Mental State Examination) was associated with higher rates of reversing sarcopenia. To conclude, our results support the dynamic nature of sarcopenia and potentially reversible factors such as higher levels of physical activity and cognitive function, which could be beneficial in the treatment of community-dwelling older adults with sarcopenia.

